# Ferulic acid production by metabolically engineered *Escherichia coli*

**DOI:** 10.1186/s40643-021-00423-0

**Published:** 2021-08-10

**Authors:** Huajun Lv, Ying Zhang, Jie Shao, Haili Liu, Yong Wang

**Affiliations:** 1 CAS Key Laboratory of Synthetic Biology, CAS Center for Excellence in Molecular Plant Sciences, Shanghai Institute of Plant Physiology and Ecology, Chinese Academy of Sciences, Shanghai, 200032 China; 2 University of Chinese Academy of Sciences, Beijing, 100049 China

**Keywords:** Ferulic acid, *E. coli*, Biosynthesis, NADPH, SAM

## Abstract

Ferulic acid (*p*-hydroxy-3-methoxycinnamic acid, FA) is a natural active substance present in plant cell walls, with antioxidant, anticancer, antithrombotic and other properties; it is widely used in medicine, food, and cosmetics. Production of FA by eco‐friendly bioprocess is of great potential. In this study, FA was biosynthesized by metabolically engineered *Escherichia coli*. As the first step, the genes *tal* (encoding tyrosine ammonia-lyase, *Rs*TAL) from *Rhodobacter sphaeroides*, *sam5* (encoding *p-*coumarate 3-hydroxylase, *Se*SAM5) from *Saccharothrix espanaensis* and *comt* (encoding Caffeic acid *O*-methytransferase, *Ta*CM) from *Triticum aestivum* were cloned in an operon on the pET plasmid backbone, *E. coli* strain containing this construction was proved to produce FA from *L*-tyrosine successfully, and confirmed the function of *Ta*CM as caffeic acid *O*-methytransferase. Fermentation result revealed JM109(DE3) as a more suitable host cell for FA production than BL21(DE3). After that the genes expression strength of FA pathway were optimized by tuning of promoter strength (T7 promoter or T5 promoter) and copy number (pBR322 or p15A), and the combination p15a-T5 works best. To further improve FA production, *E. coli* native *pntAB*, encoding pyridine nucleotide transhydrogenase, was selected from five NADPH regeneration genes to supplement redox cofactor NADPH for converting *p*-coumaric acid into caffeic acid in FA biosynthesis process. Sequentially, to further convert caffeic acid into FA, a non-native methionine kinase (MetK from *Streptomyces spectabilis*) was also overexpressed. Based on the flask fermentation data which show that the engineered *E. coli* strain produced 212 mg/L of FA with 11.8 mg/L caffeic acid residue, it could be concluded that it is the highest yield of FA achieved by *E. coli* K-12 strains reported to the best of our knowledge.

**Supplementary Information:**

The online version contains supplementary material available at 10.1186/s40643-021-00423-0.

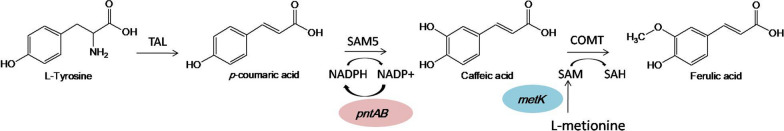

## Introduction

FA is a ubiquitous phenolic acid naturally presents in plant cell walls, mainly cross linked with polysaccharides and lignin (Harris and Trethewey 2009). It is the main effective component of *Angelica sinensis*, *Ligusticum chuanxiong* Hort., *Ferula foetida* and other traditional Chinese medicinal herbs (Liang et al. 2018). FA is an important plant antioxidant that acts as a free radical scavenger. Additionally, its antimicrobial, anti-inflammatory, anticancer, antithrombotic and other pharmaceutical activities have led to the widespread use of FA in food, cosmetics and medicine (Sgarbossa et al. 2015; Wen and Ushio 2017). Extracting FA through alkaline hydrolysis or esterase treatment of plant materials rich in FA, such as *Angelica sinensis*, *Ferula*, *Ligusticum chuanxiong *Hort., wheat and rice bran, are the main sources of commercial FA. However, these processes require costly and challenging membrane separation during downstream processing.

Plant tissue culture can provide higher yields than extraction from plant material, but this process generates pollution and there is still no industrial scale-up. Chemical synthesis of FA from vanillin via the Wittig–Horner reaction or Knoevenagel reaction offer a short producing cycle, low cost, and large output, but these approaches produce a mixture of *trans-* and *cis-*ferulic acids, which are challenging to separate.

Microbial approaches provide a promising alternative to chemical synthesis and extraction from plant sources. Products obtained through biotechnological processes from natural substrates are considered as natural for the purposes of product labeling (Serra et al. 2005). With the approval of such products by the FDA and European legislation, many studies are focused on approaches based on biotechnological methods for the production of flavors, fragrances, and pharmaceutical products (Fowler and Koffas 2009; Goris et al. 2020; Luziatelli et al. 2019). There are many reports on the heterologous biosynthesis of FA-related natural products such as *p*-coumaric acid, caffeic acid, vanillin, and curcuminoids (Braga and Faria 2020; Jendresen et al. 2015; Rodrigues et al. 2015b), but there are few papers and patents on the biosynthesis of FA (Additional file 1: Table S1). Choi et al. obtained an FA titer of 7.1  ±  1.3 mg/L via the expression of tyrosine ammonia-lyase and 4-coumarate 3-hydroxylase from *S. espanaensis*, together with *O*-methyltransferase from *Arabidopsis thaliana*. Tyrosine ammonia-lyase catalyzes the non-oxidative deamination of the primary amino acid tyrosine into *p*-coumaric acid, which is converted into caffeic acid by *p*-coumarate 3-hydroxylase. Finally, FA is biosynthesized from caffeic acid by the enzyme caffeine *O*-methyltransferase. Consequently, tyrosine is transformed into ferulic acid (Choi et al. 2011). Kang et al. expanded on this work by constructing a chromosomally engineered tyrosine over-producing strain based on *E. coli* C41(DE3) to explore the potential of FA production from simple carbon sources. After additional codon optimization of the *tal* gene, the titer of FA was increased to 196 mg/L (Kang et al. 2012).

Heterologous biosynthesis of FA in *E. coli* relies on multiple aspects, including the tuning of the expression levels of pathway enzymes, optimizing the supply of redox cofactors and precursors, and also the external addition of tyrosine.

A high gene copy number is generally desired for maximal expression, but the resulting metabolic burden can reduce the overall productivity in certain metabolic engineering applications. Similarly, choosing suitable promoter strength is also an effective way to obtain high yields. The combination of promoters and gene copy-numbers to modulate the expression levels of up and downstream pathways resulted in a remarkable 15,000-fold increase of the taxadiene titer in *E. coli* (Ajikumar et al. 2010; Jones et al. 2000; Ni et al. 2015). It is convenient to tune the expression levels of pathway genes using different combinations of plasmid replicon and promoter strengths.

In the FA biosynthesis pathway, the *p*-coumarate 3-hydroxylase, SAM5, converts *p*-coumaric acid into caffeic acid and consumes NADPH (Nicotinamide adenine dinucleotide phosphate) at the same time. Accordingly, numerous studies have shown that increasing the NADPH regeneration rate could increase both the pathway productivity and product yield. Based on a rationally engineered heterologous Entner–Doudoroff pathway that could increase the NADPH regeneration rate in *E. coli* MG1655, the product titer of the carotenoid biosynthesis pathway was increased by 97% (Martinez et al. 2008; Ng et al. 2015). The pentose phosphate pathway (PPP), the tricarboxylic acid (TCA) cycle and the transhydrogenase system constitute the main sources of NADPH (Sauer et al. 2004). The NADPH/NADP^+^ ratio in *E. coli* can be enhanced to varying degrees by overexpressing several NADPH regeneration enzymes. NADPH is required for the synthesis of the key fucosyllactose precursor—guanosine 5′-diphosphate (GDP)-*L*-fucose. Intracellular redox regeneration pathways were engineered to further enhance the production of 2- and 3-fucosyllactose (Huang et al. 2017), and these gene engineering approaches may also be effective for the heterologous biosynthesis of FA.

Additionally, the caffeic acid *O*-methyltransferase COMT consumes *S*-adenosyl-*L*-methionine (SAM), which is a ubiquitous intracellular methyl donor involved in a great number of reactions (Han et al. 2016). Increasing the intracellular SAM supply is considered a generally useful approach for improving the production of natural products (Wang et al. 2007). Methionine adenosyltransferase /SAM synthetase, which catalyzes the formation of SAM from *L*-methionine and ATP, is found in *E. coli*, baker’s yeast, *Mycobacterium smegmatis*, rat liver, bovine brain, and *Saccharomyces cerevisiae* (Han et al. 2016). Overexpression of methionine adenosyltransferase may also increase the production of FA.

In this study, we engineered *E. coli* for efficient FA production. Firstly, we constructed a *tal-sam5-comt* expression cassette using genes from three different species. The copy number and promoter strength of the FA biosynthetic pathway gene expression cassette were tuned to optimize the pathway. To further increase the yield by supplying the redox cofactor NADPH and methyl donor SAM, five NADPH regeneration enzymes and one SAM producing enzyme were individually overexpressed, resulting an increased FA titer.

## Materials and methods

### Bacterial strains, plasmids and chemicals

*E. coli* DH10B was used for plasmid construction. *E. coli* JM109(DE3) was used for the expression of heterologous genes and the production of FA. pET vectors and pCL1920-T7 were used to express multiple genes. Authentic chemical FA standards, *p*-coumaric acid and caffeic acid were purchased from Yuanye (Shanghai). All restriction enzymes and DNA ligase were purchased from New England Biolabs (Shanghai). One-step-directed cloning kit was purchased from Novoprotein (Shanghai). The polymerase chain reaction (PCR) primers are synthesized from Sangon Biotech (Shanghai) company (Additional file 1: Table S2). The *E. coli* strains and plasmids used in this study are listed in Table 1.

**Table 1:** The *E. coli* strains and plasmids used in this study

Strain and plasmid	Description	Source
JM109(DE3)	endA1 glnV44 thi-1 relA1 gyrA96 recA1 mcrB + Δ(lac-proAB) e14-[F′ traD36 proAB + lacIq lacZΔM15] hsdR17(rK-Mk +) + λ(DE3)	Lab stock
DH10B	F-*mcrA* ∆ (*mrr-hsdRMS-mcrBC*) φ80*lacZ*∆M15∆*lacX*74 *recA1endA1 araD139 (ara, leu)7697 galU galK-rpsL nupG*	Lab stock
pET21d-tal	pET21d derived, T7 Prom-tal-T7 Term	Lab stock
pET21a-sam5	pET21a derived, T7 Prom-sam5-T7 Term	This study
pET21a-comt	pET21a derived, T7 Prom-comt-T7 Term	This study
pET21d-tal-sam5	pBR322 ori, Amp resistance, T7 Prom-tal-sam5-T7 Term	This study
pET21d-tal-sam5-comt	pBR322 ori, Amp resistance, T7 Prom-tal-sam5-comt-T7 Term	This study
pJF25	pBR322 ori, Apr resistance, T7 Promoter	(Wang et al. 2013)
pHJ345	pBR322 ori, Apr resistance, T5 Promoter	This study
pHJ352	P15a ori, Apr resistance, T7 Promoter	This study
pHJ354	P15a ori, Apr resistance, T5 Promoter	This study
pBR322-T7-tal-sam5-comt	pBR322 ori, Apr resistance, T7 Prom-tal-sam5-comt-T7 Term	This study
pBR322-T5-tal-sam5-comt	pBR322 ori, Apr resistance, T5 Prom-tal-sam5-comt-T7 Term	This study
p15a-T7-tal-sam5-comt	P15a ori, Apr resistance, T7 Prom-tal-sam5-comt-T7 Term	This study
p15a-T5-tal-sam5-comt	P15a ori, Apr resistance, T5 Prom-tal-sam5-comt-T7 Term	This study
pCL1920	pSC101 ori, spectinomycin resistance, cloning vector	(Lerner and Inouye 1990)
pCL1920-T7	pSC101 ori, spectinomycin resistance, T7 Prom-MCS-T7 Term	Lab stock
pCL1920-T7-Ecicd	pSC101 ori, spectinomycin resistance, T7 Prom-Ecicd-T7 Term	This study
pCL1920-T7-Ecgnd	pSC101 ori, spectinomycin resistance, T7 Prom-Ecgnd-T7 Term	This study
pCL1920-T7-Eczwf	pSC101 ori, spectinomycin resistance, T7 Prom-Eczwf-T7 Term	This study
pCL1920-T7-EcpntAB	pSC101 ori, spectinomycin resistance, T7 Prom- EcpntAB-T7 Term	This study
pCL1920-T7-CagapN	pSC101 ori, spectinomycin resistance, T7 Prom-CagapN-T7 Term	This study

### Biosynthetic pathway construction and assembly

Restriction enzyme digestions, transformations, PCR, sodium dodecyl sulfate polyacrylamide gel electrophoresis (SDS-PAGE) analysis, and other molecular biology techniques were carried out by the standard method. The primers used are listed in Additional file 1: Table S2. Cells were grown in Luria–Bertani (LB) medium containing appropriate antibiotics at 37 °C. The working concentration of antibiotics was ampicillin (100 mg/L), apramycin (100 mg/L), kanamycin (50 mg/L), and streptomycin (50 mg/L).

The FA biosynthesis pathway was expressed from the pET21d vector. Tyrosine ammonia-lyase from *Rhodobacter sphaeroides* (encoded by *tal*; GenBank No. CP033447.1) was cloned from pET21d-*tal*. The *p*-coumaric acid 3-hydroxylase from *Saccharothrix espanaensis* (encoded by *sam5*; GenBank No. HE804045.1), and caffeic acid *O*-methyltransferase from *Triticum aestivum* (encoded by *comt*; GenBank No. EF413031.1), were codon-optimized and synthesized by GenScript Company and cloned into pET21a to construct pET21a-*sam5* and pET21a-*comt*, respectively. The nucleotide sequences of genes used in this study are listed in Additional file 1: Table S3. The whole pathway was sequentially assembled based on the *BioBrick* Assembly method using *Xba*I/*Spe*I. The *Xba*I/*Hin*dIII excised DNA fragment from pET21a-*sam5* was inserted between *Spe*I/*Hin*dIII sites of pET21d-tal to obtain plasmid pET21d-*tal-sam5*. Then the *Xba*I/*Hin*dIII excised DNA fragment from pET21a-*comt* was sequentially inserted between *Spe*I/*Hin*dIII sites of pET21d-*tal-sam5* to generate plasmid pET21d-*tal-sam5-comt*.

To construct the plasmid pHJ345 (pBR322 ori, Apr resistance, T5 promoter) with backbone pBR322-T5, we substituted the T7 promoter in the plasmid pJF25 (Wang et al. 2013) (pBR322 ori, Apr resistance, T7 promoter) with the T5 promoter sequence from pQE30 (colE1 ori, Amp resistance, T5 promoter) by fusing a 5.3-kb vector fragment amplified from pJF25 using the primer pair pro-VF/pro-VR and a 110-bp insert fragment amplified from pQE30 using the primer pair pro-T5-in-F/pro-T5-in-R.

Similarly, to construct the plasmid pHJ352 (p15a ori, Apr resistance, T7 promoter) with backbone p15a-T7, we substituted the pBR322 ori in the plasmid pJF25 (pBR322 ori, Apr resistance, T7 promoter) with the p15A sequence from pACYC184 (p15A) by fusing a 3.3 kb vector fragment amplified from pJF25 using the primer pair ori-VF/ori-VR and a 913 bp insert fragment amplified from pQE30 using the primer pair ori-p15a-inF/ori-p15a-inR.

The plasmid pHJ354(p15a *ori*, Apr resistance, T5 promoter) with backbone p15a-T7 was constructed by fusing a 4.3-kb vector fragment amplified from pHJ352 using the primer pair pro-VF/pro-VR and a 110-bp insert fragment amplified from pQE30 using the primer pair ori-p15a-inF/ori-p15a-inR.

FA biosynthesis pathways with different backbones, pBR322-T7*-tal-sam5-comt*, pBR322-T5-*tal-sam5-comt*, p15a-T7-*tal-sam5-comt* and p15a-*T5-tal-sam5-comt* were constructed by T4 ligase using *Xba*I/*Hin*dIII excised DNA fragment from pET21d-*tal-sam5-comt* as insert fragment, and *Xba*I/*Hin*dIII excised DNA fragments from pJF25, pHJ345, pHJ352, pHJ354 as vector fragments, respectively.

We modified low-copy plasmid pCL1920 (G.Lerner and Inouye 1990) to get pCL1920-T7 to co-express NADPH regeneration system with FA biosynthetic pathway. pCL1920-T7 is constructed by infusion method using two PCR products: using pCL1920 as template and primers pCL1920VF/pCL1920VR to obtain a 4.0 kb DNA fragment as vector fragment, and using pET28a as template and primers T7 operator F/ T7 operator R to obtain a 1.9-kb DNA fragment as insert fragment.

The genes *zwf, gnd, icd and pntAB* were PCR amplified from the genomic DNA of *E. coli* MG1655 and cloned into the pET28a vector. Subsequently the digested fragments were inserted into pCL1920-T7 to generate pCL1920-T7-*zwf*, pCL1920-T7-*gnd*, pCL1920-T7-*icd*, pCL1920-T7-*pntAB*, respectively. The *gapN* gene from *Clostridium. acetobutylicum* ATCC 824 (Genbank No. AE001437.1) was fused with the pCL1920-T7 backbone to generate pCL1920-T7-*gapN*. The *metK* gene from *Streptomyces spectabilis* (Genbank No. WP_144002349.1) was codon-optimized and synthesized by Genscript Company. The synthetic fragment was cloned into pET21a, and subcloned into pCL1920-T7 using *Xba*I/*Hin*dIII to generate pCL1920-T7-*metK*. All constructs were verified by Sanger sequencing (Sangon Biotech).

### Flask fermentation

The clones transformed with the indicated plasmid combinations were picked from plates and grown in LB medium at 37 ℃and 250 rpm for 12 h. On the next day, 500μL of the resulting seed culture was used to inoculate 10 mL of M9Y medium supplemented with 2% (v/v) glycerol and 1 g/L *L*-tyrosine based on a previous publication (Ni et al. 2015), in which higher specific titer was obtained by using this medium compared with LB medium or M9 minimal medium, and glycerol was suggested as more suitable carbon source than glucose. M9Y medium is modified M9 minimal salt medium containing 1 g NH_4_Cl, 6 g Na_2_HPO_4_, 3 g KH_2_PO_4_, 0.5 g NaCl, 2 mmol MgSO_4_, 0.1 mmol CaCl_2_, 0.5 g yeast extract and 1 mL trace elements (0.03 g/L H_3_BO_3_, 1 g/L thiamine, 0.94 g/L ZnCl_2_, 0.5 g/L CoCl_2_, 0.38 g/L CuCl_2_, 1.6 g/L MnCl_2,_ and 3.6 g/L FeCl_2_) per liter. IPTG was added to the cultures to a final concentration of 0.1 mM, and cultures were transferred to a shaker at 28 °C, 250 rpm for 3–5 days. Samples were collected at intervals of 24 h when needed to monitor the OD_600_ and product concentration by HPLC (high-performance liquid chromatography). All the experiments were conducted in triplicate.

### Analytical methods

Cell growth was monitored by measuring the absorbance at 600 nm (OD_600_ nm) with an UV-1200 spectrophotometer (APADA).

The products FA, *p*-coumaric acid, and caffeic acid in culture were extracted twice by an equivalent volume of ethyl acetate. The supernatant was collected and evaporated to dryness and dissolved in an equal volume methanol. The samples were analyzed by high-performance liquid chromatography on an Ultimate 3000 system (Thermo Scientific, USA) using a C18 column (Silgreen HPLC column 250  ×  4.6 mm; particle size, 5 µm) and a DAD detector at 310 nm. Compounds were separated by water (containing 0.1% acetic acid, mobile phase B) and methanol (containing 0.1% acetic acid, mobile phase C) at a flow rate of 1.0 mL/min and column temperature 30 °C under the following conditions: 80% B  +  20% C in 0–2 min, 50% B  + 50% C in 15 min, 80% B  +  20% C in 17 min, 80% B  +  20% C in 20 min. The retention time of FA, *p*-coumaric acid, and caffeic acid were 17.318 min, 16.5 min, and 12.99 min, respectively. The compounds were quantified based on calibration curves of various concentrations of standards using peak area.

### Accession numbers

The GenBank accession numbers for the nucleotide sequence of the codon-optimized *tal, sam5, comt* and *metK* genes are MW403919, MW403920, MW403921 and MW403922, respectively.

## Results

### Construction of a heterologous FA biosynthetic pathway

**Fig.1:**
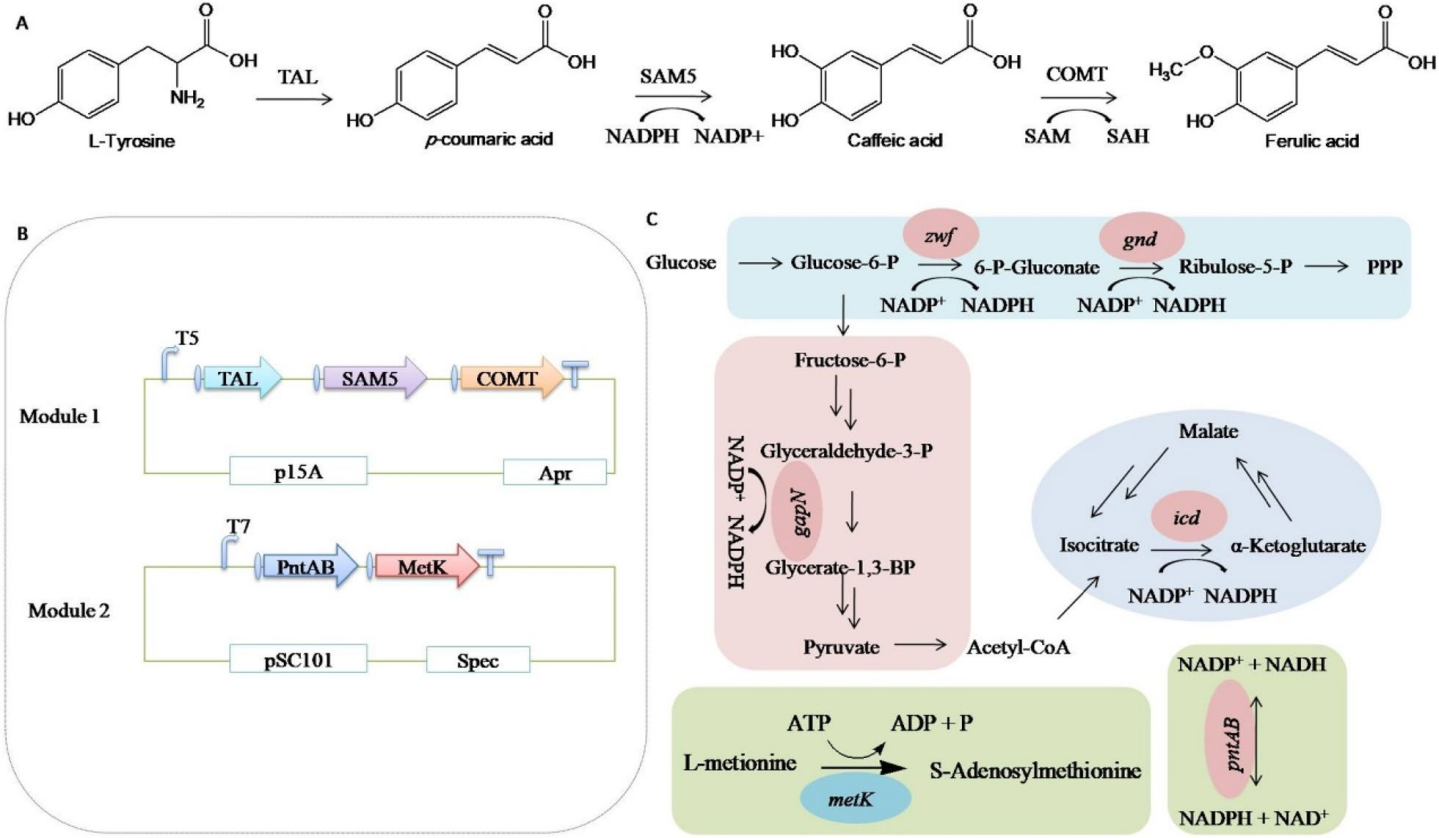
Construction of a heterologous FA biosynthesis pathway in *E. coli* (**A**). Tyrosine ammonia-lyase (TAL) catalyzes the non-oxidative deamination of the primary amino acid tyrosine into *p*-coumaric acid, which is converted into caffeic acid through hydroxylation at the 3-position of the benzyl ring by the *p*-coumarate 3-hydroxylase (SAM5). Finally, FA is biosynthesized from caffeic acid by the enzyme caffeate *O*-methyltransferase (COMT). Schematic representation of the FA biosynthesis pathway (**B**). NADPH and SAM regenerating enzymes used to increase FA production (**C**)

**Fig.2:**
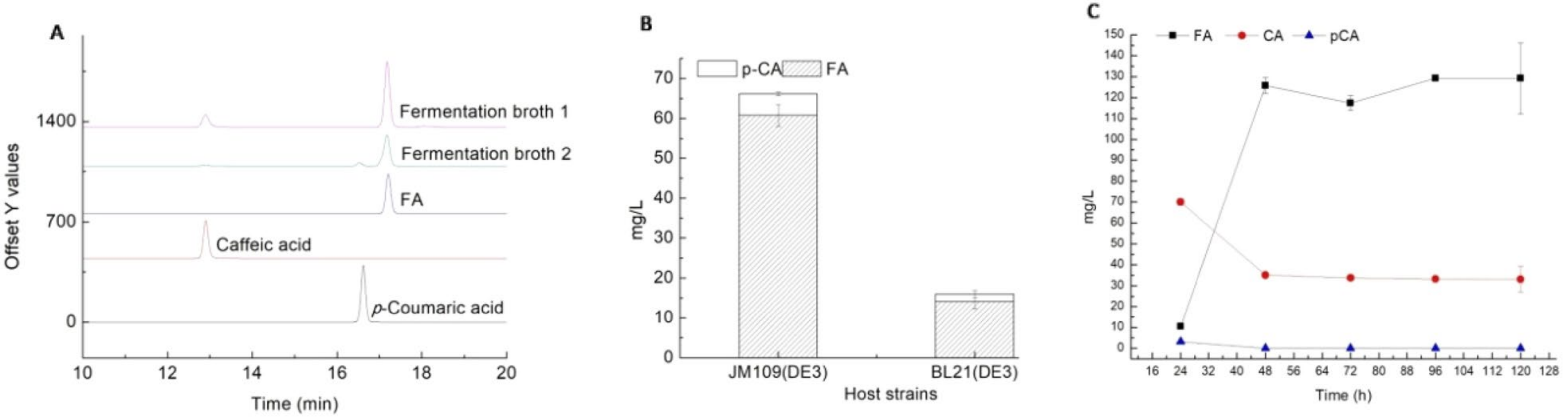
Heterologous biosynthesis of FA in *E. coli.* **A** HPLC profiles of the fermentation products and the FA, *p*-coumaric acid and caffeic acid standards. **B** FA production by strains based on *E. coli* JM109(DE3) and BL21(DE3) carrying the plasmid pBR322-T7-tal-sam5-comt. The fermentation was carried out in terrific broth (TB) medium with the addition of 1 g/L *L*-tyrosine and 2% (v/v) glycerol at 28 °C and 250 rpm for 3 days. *p-CA* *p*-coumaric acid; *FA* *trans*-ferulic acid. **C** Time course of FA production from *L*-tyrosine by *E*. *coli* JM109(DE3) T5FA in M9Y medium supplemented with 2% (v/v) glycerol and 1 g/L *L*-tyrosine at 28 °C and 250 rpm for 5 days

To construct a heterologous FA biosynthesis pathway (Fig. 1A), the *tal*, *sam5* and *comt* genes, respectively, encoding tyrosine ammonia-lyase, *p*-coumarate 3-hydroxylase, and caffeic *O*-methyltransferase from *Rhodobacter sphaeroides*, *Saccharothrix espanaensis,* and *Triticum aestivum*, respectively, were codon-optimized, synthesized, and assembled into pET21d/a or plasmid as a single operon under the control of the strong T7 promoter, resulting pBR322-T7-*tal-sam5-comt*. The construct was introduced to *E. coli* BL21(DE3) and JM109(DE3), and the recombinant *E. coli* strains were fermented in Terrific Broth (TB) medium with additional 1 g/L *L*-tyrosine and 2% (v/v) glycerol at 28 °C and 250 rpm for 3 days. FA, caffeic acid and *p*-coumaric acid were detected via HPLC analysis (Fig. 2A), but the final product FA was obtained with little *p*-coumaric acid residual in the fermentation broth. The data showed that the strain based on the *E. coli* JM109(DE3) chassis produced significantly more FA than the strain based on BL21(DE3) (60 *vs. *14 mg/L, Fig. 2B). Thus, *E. coli* JM109(DE3) was chosen as the host cell for FA production in further experiments.

The protein expression levels of *tal*, *sam5* and *comt* were very high under the control of the T7 promoter at all tested temperatures (37, 28, 22 °C), but the protein solubility was improved at lower temperatures (Additional file 1: Figure S1A–C). In order to alleviate the expression burden, we exchanged the very strong T7 promoter with the less extreme T5 promoter. The expression level of each gene was lower under the control of the T5 promoter, but the solubility at 28 °C was improved compared to the T7 promoter (Additional file 1: Figure S1D). The new expression cassette containing *tal*, *sam5* and *comt* under the control of the T5 promoter was introduced into *E. coli* JM109 (DE3), resulting in the strain T5FA. Subsequently, the recombinant *E. coli* T5FA was fermented in M9Y medium supplemented with 2% (v/v) glycerol and 1 g/L *L*-tyrosine, as described in a vanillin biosynthesis study (Ni et al. 2015). The fermentation profile showed that the titers of FA and caffeic acid increased quickly in the first 2 days, and, respectively, peaked at 130 and 33 mg/L after 48 h. By contrast, the titer of *p*-coumaric acid decreased in the first two days and was practically undetectable after 48 h (Fig. 2C).

### Pathway optimization by copy number and promoter strength tuning

**Fig. 3:**
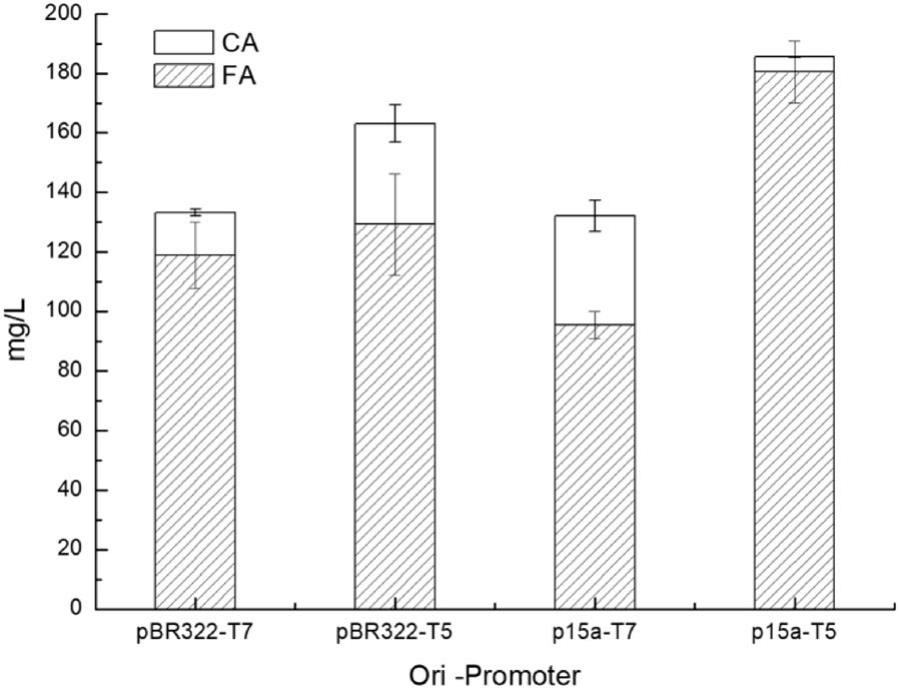
Effect of combinations of plasmid replicons (pBR322, p15a) and promoter strength (T7 promoter, T5 promoter) on FA production in host cell JM109(DE3). The fermentation was carried out in M9Y medium supplemented with 2% (v/v) glycerol and 1 g/L *L*-tyrosine at 28 °C and 250 rpm for 5 days

As mentioned above, tuning the expression levels of pathway enzymes could impact greatly on the final production, here, we replaced the medium copy number replicon pBR322 of FA biosynthesis pathway with the lower copy number replicon p15a. In order to keep the same antibiotic resistance, pET21d-tal-sam5-comt was further constructed to pBR322-T7-tal-sam5-comt, and no significant FA yields differences were observed (data not shown). Combined with promoter T7 or T5, FA biosynthesis pathway in plasmid backbones pBR322-T5, pBR322-T7, p15a-T5 and p15a-T7 (Fig. 1B), with different expression levels of pathway enzymes were obtained. Flask fermentation was carried out at the same condition as previous mentioned and the result showed that strain p15aT5 with FA biosynthesis pathway in plasmid p15a-T5-*tal-sam5-comt* works best (Fig. 3). FA production of strain p15aT5 reached 180 mg/L which is significantly higher than other strains. And there was little caffeic acid residual (~ 5 mg/L) in the fermentation, compared with strain T5FA, in which FA and caffeic acid production was 130 mg/L and 33 mg/L, respectively.

The fermentation profiles of most strains showed that the FA production peaked after 2 or 3 days, the strains co-expressing NADPH regenerating enzymes peaked later, after 4 days. We detected the production in day 3 and day 5, and showed the data of day 5 here.

### Improving FA production by NADPH regeneration enzyme

#### Further improvement of FA production by increased NADPH regeneration

The transformation of *p*-coumaric acid into caffeic acid in the FA biosynthesis pathway is catalyzed by NADPH consuming *p*-coumarate 3-hydroxylase (SAM5) (Fig. 1A).To increase NADPH availability for FA production, genes encoding NADPH regeneration enzymes (Fig. 1C), including *zwf*, *gnd*, *icd*, and *pntAB* from *E. coli* MG1655, as well as *gap*N form *Clostridium acetobutylicum* ATCC 824 (GenBank No. AE001437.1) were individually cloned into the low-copy-number vector pCL1920-T7 (~ 5 copies per cell) and introduced into JM109(DE3) together with the FA biosynthesis pathway.

The strain T5FA  +  *pntAB*, co-expressing the *pntAB* genes with the FA biosynthesis pathway, produced 192  ±  9.6 mg/L FA with 30  ±  10.0 mg/L residual caffeic acid, which was a significant improvement compared with the 140  ±  7.7 mg/L FA and 30  ±  14.8 mg/L residual caffeic acid of the control strain T5FA  +  pCL1920-T7.

**Fig. 4:**
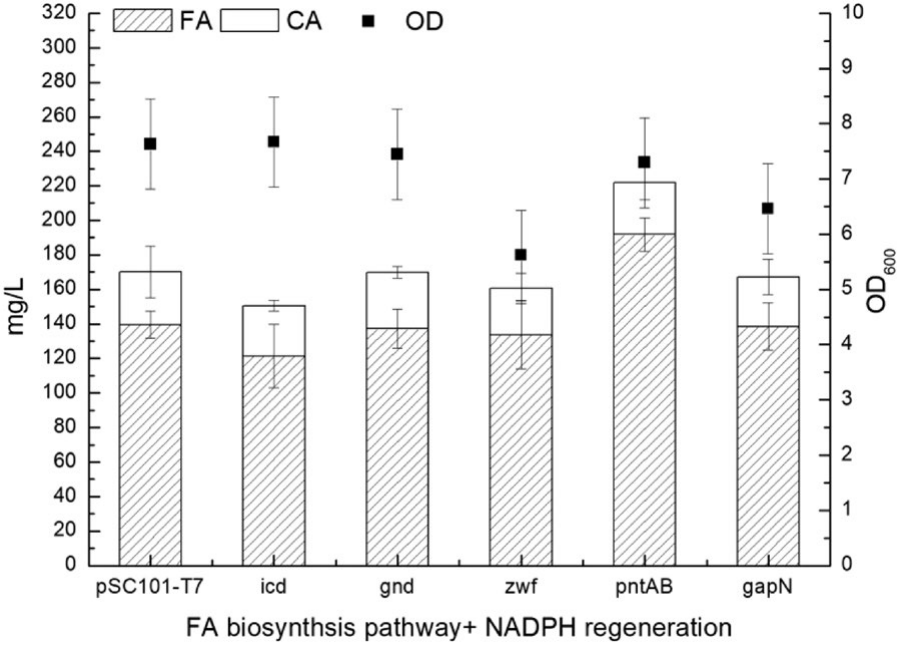
Comparison of FA biosynthesis strains co-expressed with NADPH regenerating enzymes. pSC101-T7 refers to FA biosynthesis pathway plasmid pBR322-T5-tal-sam5-comt co-expressed with plasmid pCL1920-T7 in host cell JM109(DE3); *icd* refers to FA biosynthesis pathway plasmid pBR322-T5-tal-sam5-comt co-expressed with plasmid pCL1920-T7-*Ecicd* in host cell JM109(DE3), *gnd* refers to FA biosynthesis pathway plasmid pBR322-T5-tal-sam5-comt co-expressed with plasmid pCL1920-T7- *Ecgnd* in host cell JM109(DE3), *zwf* refers to FA biosynthesis pathway plasmid pBR322-T5-tal-sam5-comt co-expressed with plasmid pCL1920-T7- *Eczwf* in host cell JM109(DE3), *pntAB* refers to FA biosynthesis pathway plasmid pBR322-T5-tal-sam5-comt co-expressed with plasmid pCL1920-T7- *EcpntAB* in host cell JM109(DE3), *gapN* refers to FA biosynthesis pathway plasmid pBR322-T5-tal-sam5-comt co-expressed with plasmid pCL1920-T7- *CagapN* in host cell JM109(DE3). The fermentation was carried out in M9Y medium supplemented with 2% (v/v) glycerol and 1 g/L *L*-tyrosine at 28 °C and 250 rpm for 5 days

Interestingly, FA production of the control strain *E. coli* T5FA  +  pCL1920-T7 (140 mg/L FA and 30 mg/L caffeic acid, Fig. 4) was marginally higher than that of *E. coli* T5FA expressing only the FA biosynthesis pathway (130 mg/L FA and 33 mg/L caffeic acid, Fig. 2c). Strains expressing the other candidate genes (*icd, zwf, gnd, gapN*) exhibited intermediate FA titers, which were higher than that of strain T5FA, but lower than that of the control strain co-transfected with the empty vector pCL1920-T7.

#### Improving FA production by non-native SAM synthetase

**Fig. 5:**
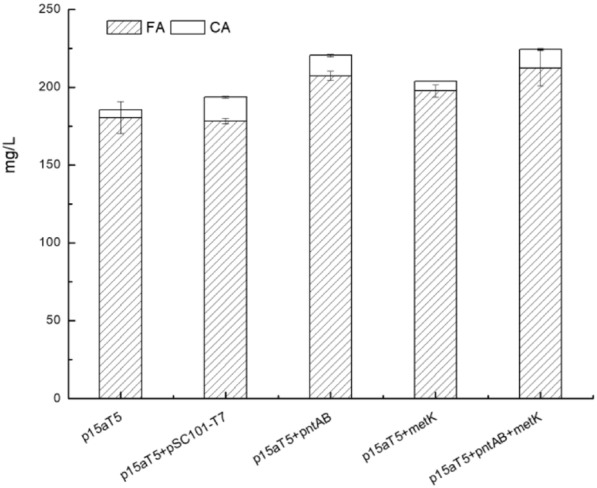
Effect of combination of NADPH regenerating enzyme *gntAB* with *S*-adenosyl-*L*-methionine synthetase *metK* in FA biosynthesis strain. p15a-T5 refers to FA biosynthesis pathway plasmid p15a-T5-tal-sam5-comt in host cell JM109(DE3); p15a-T5  +  pSC101-T7 refers to FA biosynthesis pathway plasmid p15a-T5-tal-sam5-comt co-expressed with plasmid pCL1920-T7 in host cell JM109(DE3); p15a-T5  +  pntAB refers to FA biosynthesis pathway plasmid p15a-T5-tal-sam5-comt co-expressed with plasmid pCL1920-pntAB in host cell JM109(DE3); p15a-T5  +  metK refers to FA biosynthesis pathway plasmid p15a-T5-tal-sam5-comt co-expressed with plasmid pCL1920-metK in host cell JM109(DE3); p15a-T5  +  pntAB  +  metK refers to FA biosynthesis pathway plasmid p15a-T5-tal-sam5-comt co-expressed with plasmid pCL1920-pntAB-metK in host cell JM109(DE3); The fermentation was carried out in M9Y medium supplemented with 2% (v/v) glycerol and 1 g/L *L*-tyrosine at 28 °C and 250 rpm for 5 days

To increase SAM availability for FA production, the non-native *metK* from *Streptomyces spectabilis* (Genbank No. WP_144002349.1) was cloned and fused downstream of *pntAB*, forming a bicistronic structure with *pntAB* under the control of T7 promoter on the low-copy vector pCL1920-T7 (~ 5 copies per cell), and introduced into JM109(DE3) together with FA biosynthesis pathway p15a-T5*-tal-sam5-comt*. The strain p15aT5  +  *pntAB*  +  *metK* co-expressing *pntAB* gene and *metK* gene with FA biosynthesis pathway p15aT5*-tal-sam5-comt* could increase the production significantly compared with the control strain p15aT5  +  pCL1920-T7 (Fig. 5), the production was 212.5  ±  11.4 mg/L FA and 11.9  ±  0.5 mg/L caffeic acid vs 178.3  ±  1.8 mg/L FA and 15.4  ±  0.6 mg/L; but no significant difference with strain p15aT5  +  *pntAB* (207.3  ±  2.9 mg/L FA and 13.0  ±  0.7 mg/L caffeic acid). The addition of *L*-methionine during fermentation process decreased FA production surprisingly (Additional file 1: Table S4).

## Discussion

### Construction of a heterologous FA biosynthesis pathway

The exogenous genes *tal* (Genbank No. CP033447.1), *sam5* (Genbank No. HE804045.1) and *comt* (Genbank No. EF413031.1) comprising FA biosynthesis pathway were selected based on previous studies (Berner et al. 2006; Ma and Xu 2008; Watts et al. 2004). Jendresen et al. reported that highly efficient tyrosine ammonia-lyases (TALs) from diverse origins enable enhanced production of aromatic compounds in bacteria and *Saccharomyces cerevisiae* (Jendresen et al. 2015). The *tal* gene (Genbank No. CP033447.1) performed well in our previous study on the de novo biosynthesis of resveratrol in *E. coli* (Wang et al. 2014), and the expression of the FA biosynthesis pathway using BL21(DE3) as the host cell resulted in *p*-coumaric acid accumulation (Fig. 2A). Rodrigues et al. compared *p-*coumarate 3-hydroxylase from *Saccharothrix espanaensis* (*sam5*) and *Rhodopseudomonas palustris* (CYP199A2) for the conversion of *p*-coumaric acid produced from tyrosine into caffeic acid in *E. coli*. The CYP199A2 enzyme was more active, but it requires two redox partners, which may complicate its heterologous expression (Haslinger and Prather 2020; Rodrigues et al. 2015a). COMT from *A. thaliana* (GenBank No. AY062837) was previously used to catalyze caffeic acid to FA in several reports (Choi et al. 2011; Heo et al. 2017). Here we tested COMT from wheat (*Triticum aestivum* L. *cv*. H4564, GenBank No. EF413031.1); this enzyme was named TaCM in original literature, and was proposed to methylate phenol substrates containing aldehyde, flavonoid and CoA moieties. Thus it may have a broad substrate preferences, and can not only involves in converting caffeic acid to ferulic acid and 5-hydroxyferulic acid to sinapic acid, but also in the conversion of caffeoyl-CoA to feruloyl-CoA, and 5-hydroxyferuloyl-CoA to sinapoyl-CoA (Ma and Xu 2008). Notably the protein sequence of TaCM has 100% identity with TaOMT2 (Genbank No. DQ223971), which showed the highest activity specifically to tricetin in substrate preference test against a number of phenolic compounds, and was proposed to accept caffeic acid and 5-hydoxy-ferulic acid as substrates (Zhou et al. 2006, 2009). Here, we used TaCM in the FA biosynthesis pathway and confirmed its ability to convert caffeic acid into FA. To our best knowledge, this is the first report of the caffeic acid *O*-methyltransferase activity of TaCM/TaOMT2.

*E. coli* BL21(DE3) is generally a more effective expression host than JM109(DE3), and the latter is a high acetate producer (Shiloach et al. 1996), while acetate accumulation can reduce the growth rate and recombinant protein synthesis (Noronha et al. 2000). In a study by Kang et al. *E. coli* C41(DE3) [mutant derivative of BL21(DE3)] was engineered as a tyrosine over-producing chassis for the biosynthesis of phenylpropanoic acid (Kang et al. 2012). Similarly, Huang et al. used *E. coli* BW25113 (mutant derivative of *E. coli *K-12) for the biosynthesis of caffeic acid (Huang et al. 2013; Lin and Yan 2012). In this study, the heterologous biosynthetic pathway under the control of the strong T7 promoter was tested in *E. coli* JM109(DE3) and BL21(DE3), and the results showed that it could convert *L*-tyrosine into FA, with a small amount of residual caffeic or *p*-coumaric acid (Fig. 2a). Interestingly, the FA yields were higher when using JM109(DE3) as the host strain than with BL21(DE3), so JM109(DE3) was selected as heterologous expression host cell for further experiments.

To increase the FA titer, we firstly attempted to improve soluble protein expression using the T7 promoter by decreasing the temperature form 37–22 °C. SDS-PAGE analysis showed that TAL and SAM5 were mostly expressed in the form of inclusion bodies, and their solubility was slightly improved at the lower temperature (Additional file 1: Figure S1A–C). We next replaced the strong T7 promoter with the weaker T5 promoter, and protein expression was changed greatly. Although the total protein expression level decreased, the solubility was improved, especially for TAL and SAM5. Based on this, we replaced the T7 promoter in the FA expression cassette with the T5 promoter, and the FA titer in M9Y medium increased to 130 mg/L (Fig. 2C). This result suggests that the FA yield could be optimized by tuning the gene expression to an appropriate level.

### Pathway optimization by copy number and promoter strength tuning

Using different combinations of plasmid replicons and promoter strengths is convenient to tune the expression levels of pathway genes. Modulating diverse expression level of up- and downstream pathways of taxadiene synthesis resulted in a remarkable 15,000-fold increase of taxadiene titer in an engineered *E. coli* strain. Here our results of different plasmid replicon (p15a, about 10 copies; pBR322, about 20 copies) and promoter strength (T7, the relative strength is 4.97; T5, the relative strength is 2) combinations showed strain p15a-T5 yield the highest FA titer, 180.5  ±  10 mg/L FA, 5  ±  0.1 mg/L caffeic acid mg/L. The following two were strain pBR322-T5 and pBR322-T7, and the production of strain p15a-T7 was the lowest. The order of the corresponding replicon copies and promoter strength combinations is 10–2 > 20–2 > 20–4.97 > 10–4.97, FA titer decline in turn. The numbers refers to replicon copies and promoter strength in Ajikumar’s work (Ajikumar et al. 2010). More work is needed for even refined modulation, like screening for combinations of promoters and terminators, modular controlling via different promoters, using RBS libraries that covered a broad range of translational initiation rates, and so on (Zhang and Hong 2020), and it is becoming a universal approach to achieve high yield for heterologous biosynthesis.

It is worth noting that, production of strain pBR322-T7 (118.8  ±  11 mg/L FA, 14.4  ±  1.0 mg/L caffeic acid) is higher than that previous fermentation carried in TB, indicating culture medium composition may affect the yield.

### Improving FA production by NADPH regeneration enzyme

In the FA biosynthetic pathway, *p*-coumarate 3-hydroxylase (SAM5) consumes NADPH. Thus, the overexpression of heterologous pathway can greatly affect the intracellular redox homeostasis. The overexpression or introduction of a heterologous cofactor regeneration system is an important strategy for engineering redox homeostasis, and this approach has been successfully used for the biosynthesis of many NAD(P)-dependent products (Liu et al. 2018; Zhao et al. 2017). Here, we tested five NADPH regenerating enzymes that were proved to increase the NADPH/NADP^+^ ratio in *E. coli* (Huang et al. 2017), covering almost all NADPH regeneration systems for *E. coli*. The glucose-6-phosphate dehydrogenase ZWF and 6-phosphogluconate dehydrogenase GND are involved in the PP Pathway. Similarly, the glyceraldehyde-3-phosphate dehydrogenase GapN from *Clostridium acetobutylicum* ATCC 824 is involved in the glycolysis pathway, and the isocitrate dehydrogenase ICD participates in the TCA cycle. By contrast, the transhydrogenase PntAB catalyzes the transfer of reducing power from NADH to NADP^+^ (Fig. 1C). The strain co-expressing the *pntAB* genes with the FA biosynthesis pathway (T5FA  +  *pntAB*) exhibited a significant increase of FA production (191.9  ±  9.6 mg/L; Fig. 4). However, the other NADPH regenerating enzymes showed no effect on the FA titer. Similarly, *pntAB* overexpression to mitigate the redox imbalance significantly increased the product titer and yield in the biosynthesis of shikimic and glycolic acid in *E. coli* (Cabulong et al. 2019; Cui et al. 2014), as well as *L*-lysine, acetic and succinic acid in *Corynebacterium glutamicum* (Kabus et al. 2007; Yamauchi et al. 2014). Sauer et al. found that PntAB produced 35–45% of the NADPH consumed during the exponential batch growth phase on glucose, while the pentose phosphate pathway and isocitrate dehydrogenase contributed 35–45% and 20–25%, respectively (Sauer et al. 2004). Based on this, PntAB contributed the most among the five tested enzymes, which may explain why the strain co-expressing the *pntAB* genes with the FA biosynthesis pathway showed a significant increase of the product titer, which was not observed with the other tested genes. However, glycerol was used as the carbon source in this work, and the effect may be different if glucose is used in the case of the other NADPH regenerating enzymes, especially *zwf* and *gnd*, which are involved in the pentose phosphate pathway.

We noted that the FA production of the control strain *E. coli* T5FA  +  pCL1920-T7 (140 mg/L FA and 30 mg/L caffeic acid, Fig. 4) was slightly higher than that of *E. coli* T5FA, which carries only FA biosynthesis pathway plasmid(130 mg/L FA and 33 mg/L caffeic acid, Fig. 2C). The product titers of the strains expressing the other candidate genes (*icd, zwf, gnd, gapN*) were also lower than that of the control strain. As an amino glycoside antibiotic, spectinomycin binds to the bacterial 30S ribosomal subunit and blocks protein synthesis. In the strain carrying the pCL1920 plasmid, the protein synthesis maybe affected due to the addition of spectinomycin to the culture medium, which may reduce the burden of protein synthesis, and improve the yield of FA. Our results also showed that the strain co-transfected with a plasmid carrying a spectinomycin resistance gene exhibited better growth and FA production than the strains without this plasmid.

### Improving FA production by non-native SAM synthetase

The transformation of caffeic acid into FA consumes *S*-adenosylmethionine (SAM) at the same time (Fig. 1). Similar as redox cofactor NADPH, SAM is a ubiquitous intracellular methyl or methylate donor, previous work reported improving heterologous polyketide production in *E. coli* by overexpression of an non-native *S*-adenosylmethionine synthetase (*SsmetK*) gene (Wang et al. 2007). Increased intracellular SAM availability is beneficial to increase FA production in theory. Endogenous *S*-adenosylmethionine synthetase gene *metK* (SAMs, catalyzing the reaction of ATP and *L*-Methionine to form SAM, Fig. 1C) in *E. coli* is inhibited by *L*-methionine (Zocchi et al. 2003), for this reason, we chose the non-native *metK* from *Streptomyces spectabilis* and tested in our heterologous biosynthesis pathway.

The FA production of strain p15aT5  +  *metK*, strain p15aT5  +  *gntAB * +  *metK*, and strain p15aT5  +  *gntAB* is significantly higher than the control strain p15aT5  +  pCL1920-T7, the highest one is p15aT5  +  *gntAB*  +  *metK*, producing 212.5  ±  11.4 mg/L FA with 11.9  ±  0.5 mg/L caffeic acid residue. Caffeic acid accumulation was lowest in strain p15aT5  +  *metK* among these strains, which may attribute to supplement of SAM. Compared with strain T5FA  +  *pntAB* (191.9  ±  9.6 mg/L FA and 30  ±  10 mg/L caffeic acid), strain p15aT5  + *gntAB* (207.4  ±  2.9 mg/L FA and 13  ±  0.7 mg/L caffeic acid) produced more FA and left less caffeic acid residual, which is consistent with the expression level optimization objective. While there is no significant difference of FA production between these three strains (Fig. 5), which means that the effect of gene *metK* on FA production is almost equal with gene *pntAB*. The strain p15aT5  +  *pntAB * +  *metK* showed no increase of the FA production further, not as expected a possible superimposed promotion effect. We also tried adding *L*-methionine in culture medium directly, but resulted in decreased FA production (Additional file 1: Table S4). Kunjapur et al. reported deletion of *metJ* coupled with expression of feedback-desensitized variants of metA* and cysE*, genes involved in methionine biosynthesis, improved de novo vanillate titers by 33% in an engineered *E. coli* K-12 MG1655 strain RARE that serves as a platform for aromatic aldehyde biosynthesis. While overexpression of *mtn* and *luxS*, genes involved in *S*-adenosylhomocysteine (SAH) recycling improved de novo vanillate titers by 25%, it is supposed to work by the mechanism of increasing SAM availability, since SAH is a potent inhibiter of SAM-dependent methyltransferases. And vanillate production improved further upon supplementation with methionine (Kunjapur et al. 2016). So, it seems that methylation improvement in biosynthetic pathway in *E. coli* could be achieved by methods of improving SAM availability like introducing heterologous methionine adenosyltransferase (*SsmetK*), engineering methionine biosynthesis related genes (*metJ*, metA* and cysE*) and SAH recycling related genes (*mtn* and *luxS*). As pointed out in Kunjapur et al.’s work, adding methionine to *E. coli* decreases flux in the Met and SAM biosynthetic pathway due to feedback inhibition of MetA. The effect of supplying methionine exogenously may vary from case to case. SAM availability in FA biosynthesis may improve and gain increased FA titer by combining all these strategies in future work. So, the highest FA titer in our work is 212 mg/L, although the FA titer of Kang et al 2012 is 196 mg/L in 36 h, and our data are 212 mg/L in 5 days, it seems that the productivity of the former is better, but the carbon supplement of the former is much higher and the host cell is BL21(DE3) (Additional file 1: Table S1). In parallel with our work, Rodrigues et al. reported even higher FA production (257 mg/L) in their work of curcuminoids biosynthesis in *E. coli* BL21(DE3) recently. They constructed TAL from *Rhodotorula glutinis* and C3H/SAM5 from *S. espanaensis* in pCDF-duet backbone (CloDF13 *ori*, PT7*lac*), and COMT from *A. thaliana* in pRSF-duet backbone (RSF1030 *ori*, PT7*lac*) simultaneously. Except for different source of TAL and COMT with our work, BL21(DE3) was chosen as host cell and two plasmids were used to construct FA biosynthesis pathway in their work (Rodrigues et al. 2020). The genes they chose were reported as most the efficient ones, while gene *tal* and gene *comt* were not the same version as those used in our work, and FA biosynthesis pathway was constructed in a combination of two plasmids, pCDF-duet_TAL (20–40 copies, PT7*lac*) and pRSF-duet_C3H_COMT (10 copies, PT7*lac*). Both the plasmid copy number and promoter strength are higher than the ones used in our work, but reached a balance in their work, although introduction of multiple plasmids is inconvenient for follow-up operations, indicating FA production could be further improved by combined application of two strategies.

In this study, we constructed a heterologous FA biosynthesis pathway by introducing genes from three different species, and the caffeic acid *O*-methyltransferase activity of TaCM was shown for the first time. FA production was tested in two common *E. coli* strains, and using JM109(DE3) as the host strain resulted in a higher FA yield than using BL21(DE3). Tuning down the promoter strength of the expression cassette significantly increased the final FA titer, so we continued to optimize the pathway expressing level using combinations of different replicons and promoter strengths, which further increased FA production. To further increase the production, the endogenous NADPH regeneration genes *pntAB *and the heterologous SAM formation enzyme *SsmetK* were used to improve the supply of the key cofactors NADPH and SAM, which greatly increased the FA titer from 130 to 212 mg/L. Further studies are needed to test for synergistic effects of simultaneous NADPH and SAM supplementation, and relevant methods such as increasing methionine biosynthesis and SAH recycling to improve SAM availability can be adopted. Strategies that can more finely modulate the flux of pathway metabolites, such as splitting the FA biosynthesis pathway into two modules and regulating each module separately, may unlock greater productivity of the host cells. Based on all these efforts, the widely used heterologous expression host BL21(DE3) with glucose as carbon source may still show good productivity in the heterologous biosynthesis of FA. A tyrosine over-producing strain or chromosomal integration of the biosynthesis pathway can also tested to reduce the fermentation cost. This study lacks fed-batch fermentation data, but the shake-flask results were comparable with the literature, indicating good application prospects. Our work offers a basis for further studies on the heterologous biosynthesis of phenolic natural products.

### Supplementary Information

**Additional file 1: Table S1.** Efforts for producing FA in *E. coli*. **Table S2.** The polymerase chain reaction (PCR) primers used in this study. **Table S3.** Nucleotide sequences of genes used in this study. **Table S4.** Effect of adding *L*-Methionine in FA biosynthetic pathway. **Figure S1.** Sodium dodecyl sulfate polyacrylamide gel electrophoresis results of TAL, SAM5 and COMT expression.

## Data Availability

All datasets used and analyzed in the current study are available from the corresponding author on reasonable request.

## Abbreviations

FA: *p*-Hydroxy-3-methoxycinnamic acid
NADPH: Nicotinamide adenine dinucleotide phosphate
SAM: *S*-Adenosyl-*L*-methionine
HPLC: High-performance liquid chromatography
