# Supplementary material for: Ferulic acid production by metabolically engineered Escherichia coli
Source: Bioresour Bioprocess. 2021 Aug 10;8(1):70. doi: 10.1186/s40643-021-00423-0 (PMC10992898; doi:10.1186/s40643-021-00423-0)
Supplement: Supplementary file 1 — Additional file 1: Table S1. Efforts for producing FA in E. coli. Table S2. The polymerase chain reaction (PCR) primers used in this study. Table S3. Nucleotide sequences of genes used in this study. Table S4. Effect of adding L-Methionine in FA biosynthetic pathway. Figure S1. Sodium dodecyl sulfate polyacrylamide gel electrophoresis results of TAL, SAM5 and COMT expression. [file 40643_2021_423_MOESM1_ESM.docx]

Supplementary materials

**Journal name** *Bioresources* and *Bioprocessing*

**Manuscript Title:** Ferulic Acid production by metabolically engineered *Escherichia coli*

Huajun Lv^1^, Ying Zhang^1,2^, Jie Shao^1,2^, Haili Liu^1^, Yong Wang^*1^

1 *CAS Key Laboratory of Synthetic Biology, CAS Center for Excellence in Molecular Plant Sciences, Shanghai Institute of Plant Physiology and Ecology, Chinese Academy of Sciences, Shanghai 200032, China*

2 *University of Chinese Academy of Sciences, Beijing 100049, China*

*Corresponding author.

E-mail address: [yongwang@cemps.ac.cn](mailto:yongwang@cemps.ac.cn) Tel/Fax:86-21-54924295

These authors contributed equally: Huajun Lv, Ying Zhang.

**Table S1** Efforts for producing FA in *E. coli*

| FA titer | Strains | Gene source | Fermentation conditions | Reference |
| --- | --- | --- | --- | --- |
| 7.1 mg/L | *E. coli* C41(DE3) | *tal*, *sam5* from *Saccharothrix espanaensis* and *comt* from *Arabidopsis thaliana* | Recombinant *E. coli* C41 (DE3) strains harboring FA biosynthetic pathway plasmid (pBR322, T7) was inoculated into LB medium. The culture was grown at 37 °C to optical density at OD_600_ of 0.6, IPTG was added to the final concentration of 1 mM, after incubated in LB medium for 5 h, the medium was changed into modified M9 medium (40 g/L glucose 25 g/L CaCO_3_, pH 6.8–7.0), incubated for 36 hours at 26 °C. | (Choi et al., 2011) |
| 196 mg/L | Engineered tyrosine overproducing *E. coli* C41(DE3) | *tal*, *sam5* from *S. espanaensis* and *comt* from *Arabidopsis thaliana* | The same incubation condition as above. | (Kang et al., 2012). |
| 156 mg/L | *E. coli* BL21(DE3) | *tal*, *sam5* from *Saccharothrix espanaensis* and codon-optimized *comt* from *Arabidopsis thaliana* | *E. coli* BL21(DE3) harboring FA biosynthetic pathway plasmid (p15A, T7) was grown in LB medium with an additional 2 g/L l-tyrosine at 37 °C. After the OD600 reached 0.5- 0.6, IPTG was added to the cultures to a final concentration of 0.2 mM, and cultures were transferred to a gyratory shaker at 26 °C for 3 days. | (Ni et al., 2015) |
| 28.8  mg/L | *E. coli* BL21(DE3) | *tal*, *sam5* from *Saccharothrix espanaensis* and *comt* from *Medicago sativa* | *E. coli* BL21(DE3) harboring FA biosynthetic pathway plasmid (p15A, T7) was grown in LB medium with an additional 2 g/L L-tyrosine at 37 °C. After the OD600 reached 0.5- 0.6, IPTG was added to the cultures to a final concentration of 0.2 mM, and cultures were transferred to a gyratory shaker at 26 °C for 3 days. | (Wang et al., 2015) |
| 257.3 mg/L | *E. coli* BL21(DE3) | Codon-optimized *tal* from *Rhodotorula glutinis*, codon-optimized *c3h* from *Saccharothrix espanaensis*, codon-optimized *comt* from *Arabidopsis thaliana* | *E. coli* carrying pCDFduet_TAL and pRSFduet_C3H_COMT were grown at 37 °C in 50 mL LB from an OD600 of 0.1(≈ 2% inoculums volume) up to 0.9. The protein expression was induced with IPTG (0.1 mM) and the culture was then incubated for 5 h at 26 °C. Next, the cells were harvested by centrifugation, suspended, and incubated at 26 °C for 63 h in 50 mL M9 medium. Tyrosine (3 mM) and IPTG (0.1 mM) were added at time 0 of induction in modified M9 medium (glucose 40 g/L, trace elements and vitamins). | (Rodrigues et al., 2020) |

**Table S2** The polymerase chain reaction (PCR) primers used in this study

| Primers | Sequence |
| --- | --- |
| T7 operatorF: | CGGGAATTCGCGCAAAAAACCCCTCAAG |
| T7 operatorR: | CTTTACTAAGCTGACGATAGTCATGCCCCG |
| pCL1920VF: | GACTATCGTCAGCTTAGTAAAGCCCTCGCTAG |
| pCL1920VR: | GTTTTTTGCGCGAATTCCCG |
| pro-VF | AGATCTCGATCCTCTACGC |
| pro-VR | TCTAGAAATAATTTTGTTTAACTTTAAG |
| pro-T5-in-F | CAAAATTATTTCTAGAATAGTTAATTTCTCCTC |
| pro-T5-in-R | GTAGAGGATCGAGATCTAAATCATAAAAAATTTATTTG |
| ori-p15a-inF: | CAAAAGCACCGCCGGACATCAGCGCTAGCGGAGTGTATACTG |
| ori-p15a-inR | CATGACTAACATGAGAATTACAACTTATATCGTATG |
| ori-VF: | CATACGATATAAGTTGTAATTCTCATGTTAGTCATG |
| ori-VR | CAGTATACACTCCGCTAGCGCTGATGTCCGGCGGTGCTTTTG |
| ECicd F | CCAAGCTTACTAGTTTACATGTTTTCGATG |
| ECicd R | CATGCCATGGAAAGTAAAGTAGTTG |
| ECgnd F | CCAAGCTTACTAGTTTAATCCAGCCATTCG |
| ECgnd R | GGAATTCCATATGTCCAAGCAACAGATC |
| ECzwfF | CCCAAGCTTACTAGTTTACTCAAACTCATTCCAG |
| ECzwfR | CATGCCATGGCGGTAACGCAAACAG |
| pntABHindF | CCCAAGCTTACTAGTTTACAGAGCTTTCAGGATTG |
| pntABNcoR | CATGCCATGGAAGGGAATATCATG |
| gapNF | CTTTAAGAAGGAGATATACATGTTTGAAAATATATCATCAAATGGAG |
| gapNR | GTGCGGCCGCAAGCTTGTCGATTATAGGTTTAAAACTATTGATTTATG |
| GapN-V F | CATAAATCAATAGTTTTAAACCTATAATCGACAAGCTTGCGGCCGCAC |
| GapN-VR | CTCCATTTGATGATATATTTTCAAACATGTATATCTCCTTCTTAAAG |

**Table S3** Nucleotide sequences of genes used in this study

| Gene | Sequence |
| --- | --- |
| *tal* | atggaaatgctggcgatgagcccgccgaaaccggcggtggaactggatcgccatattgatctggatgaagcgcatagcgtggcgagcggcggcgcgcgcattgtgctggcgccgccggcgcgcgatcgctgccgcgcgagcgaagcgcgcctgggcgcggtgattcgcgaagcgcgccatgtgtatggcctgaccaccggctttggcccgctggcgaaccgcctggtgagcggcgaaaacgtgcgcaccctgcaggcgaacctggtgcatcatctggcgagcggcgtgggcccggtgctggattggaccaccgcgcgcgcgatggtgctggcgcgcctggtggcgattgcgcagggcgcgagcggcgcgagcgaaggcaccattgcgcgcctgattgatctgctgaacagcgaactggcgccggcggtgccgatgcgcggcaccgtgggcgcgagcggcgatctgaccccgctggcgcatatggtgctgtgcctgcagggccgcggcgattttctggatcgcgatggcacccgcctggatggcgcggaaggcctgcgccgcggccgcctgcagccgctggatctgagccatcgcgatgcgctggcgctggtgaacggcaccagcgcgatgaccggcattgcgctggtgaacgcgcatgcgtgccgccatctgggcaactgggcggtggcgctgaccgcgctgctggcggaatgcctgggcggccgcaccgaagcgtgggcggcggcgctgagcgatctgcgcccgcatccgggccagaaagatgcggcggcgcgcctgcgcgcgcgcgtggatggcagcgcgcgcgtggtgcgccatgtgattgcggaacgccgcctgggcgcgagcgatattggcaccgaaccggaagcgggccaggatgcgtatagcctgcgctgcgcgccgcaggtgctgggcgcgggctttgataccctggcgtggcatgatcgcgtgctgaccattgaactgaacgcggtgaccgataacccggtgtttccgccggatggcagcgtgccggcgctgcatggcggcaactttatgggccagcatgtggcgctgaccagcgatgcgctggcgaccgcggtgaccgtgctggcgggcctggcggaacgccagattgcgcgcctgaccgatgaacgcctgaaccgcggcctgccgccgtttctgcatcgcggcccggcgggcctgaacagcggctttatgggcgcgcaggtgaccgcgaccgcgctgctggcggaaatgcgcgcgaccggcccggcgagcattcatagcattagcaccaacgcggcgaaccaggatgtggtgagcctgggcaccattgcggcgcgcctgtgccgcgaaaaaattgatcgctgggcggaaattctggcgattctggcgctgtgcctggcgcaggcggcggaactgcgctgcggcagcggcctggatggcgtgagcccggcgggcaaaaaactggtgcaggcgctgcgcgaacagtttccgccgctggaaaccgatcgcccgctgggccaggaaattgcggcgctggcgacccatctgctgcagcagagcccggtgtaa |
| *sam5* | atgaccatcacctctccggcgccggcgggtcgtctgaacaacgttcgtccgatgaccggtgaagaatacctggaatctctgcgtgacggtcgtgaagtttacatctacggtgaacgtgttgacgacgttaccacccacctggcgttccgtaactctgttcgttctatcgcgcgtctgtacgacgttctgcacgacccggcgtctgaaggtgttctgcgtgttccgaccgacaccggtaacggtggtttcacccacccgttcttcaaaaccgcgcgttcttctgaagacctggttgcggcgcgtgaagcgatcgttggttggcagcgtctggtttacggttggatgggtcgtaccccggactacaaagcggcgttcttcggtaccctggacgcgaacgcggaattctacggtccgttcgaagcgaacgcgcgtcgttggtaccgtgacgcgcaggaacgtgttctgtacttcaaccacgcgatcgttcacccgccggttgaccgtgaccgtccggcggaccgtaccgcggacatctgcgttcacgttgaagaagaaaccgactctggtctgatcgtttctggtgcgaaagttgttgcgaccggttctgcgatgaccaacgcgaacctgatcgcgcactacggtctgccggttcgtgacaaaaaattcggtctggttttcaccgttccgatgaactctccgggtctgaaactgatctgccgtacctcttacgaactgatggttgcgacccagggttctccgttcgactacccgctgtcttctcgtctggacgaaaacgactctatcatgatcttcgaccgtgttctggttccgtgggaaaacgttttcatgtacgacgcgggtgcggcgaactctttcgcgaccggttctggtttcctggaacgtttcaccttccacggttgcacccgtctggcggttaaactggacttcatcgcgggttgcgttatgaaagcggttgaagttaccggtaccacccacttccgtggtgttcaggcgcaggttggtgaagttctgaactggcgtgacgttttctggggtctgtctgacgcgatggcgaaatctccgaactcttgggttggtggttctgttcagccgaacctgaactacggtctggcgtaccgtaccttcatgggtgttggttacccgcgtatcaaagaaatcatccagcagaccctgggttctggtctgatctacctgaactcttctgcggcggactggaaaaacccggacgttcgtccgtacctggaccgttacctgcgtggttctcgtggtatccaggcgatcgaccgtgttaaactgctgaaactgctgtgggacgcggttggtaccgaattcgcgggtcgtcacgaactgtacgaacgtaactacggtggtgaccacgaaggtatccgtgttcagaccctgcaggcgtaccaggcgaacggtcaggcggcggcgctgaaaggtttcgcggaacagtgcatgtctgaatacgacctggacggttggacccgtccggacctgatcaacccgggtacctaa |
| *comt* | atgggttctatcgcggcgggtgcggacgaagacgcgtgcatgtacgcgctgcagctggtttcttcttctatcctgccgatgaccctgaaaaacgcgatcgaactgggtctgctggaaaccctgatggcggcgggtggtaaattcctgaccccggcggaagttgcggcgaaactgccgtctgcggcgaacccggaagcgccggacatggttgaccgtatgctgcgtctgctggcgtcttacaacgttgtttcttgccgtaccgaagaaggtaaagacggtcgtctgtctcgtcgttacggtgcggcgccggtttgcaaatacctgaccccgaacgaagacggtgtttctatgtctgcgctggcgctgatgaaccaggacaaagttctgatggaatcttggtactacctgaaagacgcggttctggacggtggtatcccgttcaacaaagcgtacggtatgtctgcgttcgaataccacggtaccgacccgcgtttcaaccgtgttttcaacgaaggtatgaaaaaccactctatcatcatcaccaaaaaactgctggaatcttacaaaggtttcgaaggtctgggtaccctggttgacgttggtggtggtgttggtgcgaccgttgcggcgatcaccgcgcactacccgaccatcaaaggtatcaacttcgacctgccgcacgttatctctgaagcgccgccgttcccgggtgttacccacgttggtggtgacatgttccagaaagttccgtctgcggacgcgatcctgatgaaatggatcctgcacgactggtctgacgaacactgcgcgaccctgctgaaaaactgctacgacgcgctgccggcgcacggtaaagttgttctggttgaatgcatcctgccggttaacccggaagcgaccccgaaagcgcagggtgttttccacgttgacatgatcatgctggcgcacaacccgggtggtcgtgaacgttacgaacgtgaattcgaagcgctggcgaaaggtgcgggtttcgcggcgatgaaaaccacctacatctacgcgaacgcgtgggcgatcgaattcaccaaataa |
| *icd* | atggaaagtaaagtagttgttccggcacaaggcaagaagatcaccctgcaaaacggcaaactcaacgttcctgaaaatccgattatcccttacattgaaggtgatggaatcggtgtagatgtaaccccagccatgctgaaagtggtcgacgctgcagtcgagaaagcctataaaggcgagcgtaaaatctcctggatggaaatttacaccggtgaaaaatccacacaggtttatggtcaggacgtctggctgcctgctgaaactcttgatctgattcgtgaatatcgcgttgccattaaaggtccgctgaccactccggttggtggcggtattcgctctctgaacgttgccctgcgccaggaactggatctctacatctgcctgcgtccggtacgttactatcagggcactccaagcccggttaaacaccctgaactgaccgatatggttatcttccgtgaaaactcggaagacatttatgcgggtatcgaatggaaagcagactctgccgacgccgagaaagtgattaaattcctgcgtgaagagatgggggtgaagaaaattcgcttcccggaacattgtggtatcggtattaagccgtgttcggaagaaggcaccaaacgtctggttcgtgcagcgatcgaatacgcaattgctaacgatcgtgactctgtgactctggtgcacaaaggcaacatcatgaagttcaccgaaggagcgtttaaagactggggctaccagctggcgcgtgaagagtttggcggtgaactgatcgacggtggcccgtggctgaaagttaaaaacccgaacactggcaaagagatcgtcattaaagacgtgattgctgatgcattcctgcaacagatcctgctgcgtccggctgaatatgatgttatcgcctgtatgaacctgaacggtgactacatttctgacgccctggcagcgcaggttggcggtatcggtatcgcccctggtgcaaacatcggtgacgaatgcgccctgtttgaagccacccacggtactgcgccgaaatatgccggtcaggacaaagtaaatcctggctctattattctctccgctgagatgatgctgcgccacatgggttggaccgaagcggctgacttaattgttaaaggtatggaaggcgcaatcaacgcgaaaaccgtaacctatgacttcgagcgtctgatggatggcgctaaactgctgaaatgttcagagtttggtgacgcgatcatcgaaaacatgtaa |
| *zwf* | atggcggtaacgcaaacagcccaggcctgtgacctggtcattttcggcgcgaaaggcgaccttgcgcgtcgtaaattgctgccttccctgtatcaactggaaaaagccggtcagctcaacccggacacccggattatcggcgtagggcgtgctgactgggataaagcggcatataccaaagttgtccgcgaggcgctcgaaactttcatgaaagaaaccattgatgaaggtttatgggacaccctgagtgcacgtctggatttttgtaatctcgatgtcaatgacactgctgcattcagccgtctcggcgcgatgctggatcaaaaaaatcgtatcaccattaactactttgccatgccgcccagcacttttggcgcaatttgcaaagggcttggcgaggcaaaactgaatgctaaaccggcacgcgtagtcatggagaaaccgctggggacgtcgctggcgacctcgcaggaaatcaatgatcaggttggcgaatacttcgaggagtgccaggtttaccgtatcgaccactatcttggtaaagaaacggtgctgaacctgttggcgctgcgttttgctaactccctgtttgtgaataactgggacaatcgcaccattgatcatgttgagattaccgtggcagaagaagtggggatcgaagggcgctggggctattttgataaagccggtcagatgcgcgacatgatccagaaccacctgctgcaaattctttgcatgattgcgatgtctccgccgtctgacctgagcgcagacagcatccgcgatgaaaaagtgaaagtactgaagtctctgcgccgcatcgaccgctccaacgtacgcgaaaaaaccgtacgcgggcaatatactgcgggcttcgcccagggcaaaaaagtgccgggatatctggaagaagagggcgcgaacaagagcagcaatacagaaactttcgtggcgatccgcgtcgacattgataactggcgctgggccggtgtgccattctacctgcgtactggtaaacgtctgccgaccaaatgttctgaagtcgtggtctatttcaaaacacctgaactgaatctgtttaaagaatcgtggcaggatctgccgcagaataaactgactatccgtctgcaacctgatgaaggcgtggatatccaggtactgaataaagttcctggccttgaccacaaacataacctgcaaatcaccaagctggatctgagctattcagaaacctttaatcagacgcatctggcggatgcctatgaacgtttgctgctggaaaccatgcgtggtattcaggcactgtttgtacgtcgcgacgaagtggaagaagcctggaaatgggtagactccattactgaggcgtgggcgatggacaatgatgcgccgaaaccgtatcaggccggaacctggggacccgttgcctcggtggcgatgattacccgtgatggtcgttcctggaatgagtttgagtaa |
| *gnd* | atgtccaagcaacagatcggcgtagtcggtatggcagtgatgggacgcaaccttgcgctcaacatcgaaagccgtggttataccgtctctattttcaaccgttcccgtgagaagacggaagaagtgattgccgaaaatccaggcaagaaactggttccttactatacggtgaaagagtttgtcgaatctctggaaacgcctcgtcgcatcctgttaatggtgaaagcaggtgcaggcacggatgctgctattgattccctcaaaccatatctcgataaaggagacatcatcattgatggtggtaacaccttcttccaggacactattcgtcgtaatcgtgagctttcagcagagggctttaacttcatcggtaccggtgtttctggcggtgaagagggggcgctgaaaggtccttctattatgcctggtggccagaaagaagcctatgaattggtagcaccgatcctgaccaaaatcgccgccgtagctgaagacggtgaaccatgcgttacctatattggtgccgatggcgcaggtcactatgtgaagatggttcacaacggtattgaatacggcgatatgcagctgattgctgaagcctattctctgcttaaaggtggcctgaacctcaccaacgaagaactggcgcagacctttaccgagtggaataacggtgaactgagcagttacctgatcgacatcaccaaagatatcttcaccaaaaaagatgaagacggtaactacctggttgatgtgatcctggatgaagcggctaacaaaggtaccggtaaatggaccagccagagcgcgctggatctcggcgaaccgctgtcgctgattaccgagtctgtgtttgcacgttatatctcttctctgaaagatcagcgtgttgccgcatctaaagttctctctggtccgcaagcacagccagcaggcgacaaggctgagttcatcgaaaaagttcgtcgtgcgctgtatctgggcaaaatcgtttcttacgcccagggcttctctcagctgcgtgctgcgtctgaagagtacaactgggatctgaactacggcgaaatcgcgaagattttccgtgctggctgcatcatccgtgcgcagttcctgcagaaaatcaccgatgcttatgccgaaaatccacagatcgctaacctgttgctggctccgtacttcaagcaaattgccgatgactaccagcaggcgctgcgtgatgtcgttgcttatgcagtacagaacggtattccggttccgaccttctccgcagcggttgcctattacgacagctaccgtgctgctgttctgcctgcgaacctgatccaggcacagcgtgactattttggtgcgcatacttataagcgtattgataaagaaggtgtgttccataccgaatggctggattaa |
| *pntAB* | Atggaagggaatatcatgcgaattggcataccaagagaacggttaaccaatgaaacccgtgttgcagcaacgccaaaaacagtggaacagctgctgaaactgggttttaccgtcgcggtagagagcggcgcgggtcaactggcaagttttgacgataaagcgtttgtgcaagcgggcgctgaaattgtagaagggaatagcgtctggcagtcagagatcattctgaaggtcaatgcgccgttagatgatgaaattgcgttactgaatcctgggacaacgctggtgagttttatctggcctgcgcagaatccggaattaatgcaaaaacttgcggaacgtaacgtgaccgtgatggcgatggactctgtgccgcgtatctcacgcgcacaatcgctggacgcactaagctcgatggcgaacatcgccggttatcgcgccattgttgaagcggcacatgaatttgggcgcttctttaccgggcaaattactgcggccgggaaagtgccaccggcaaaagtgatggtgattggtgcgggtgttgcaggtctggccgccattggcgcagcaaacagtctcggcgcgattgtgcgtgcattcgacacccgcccggaagtgaaagaacaagttcaaagtatgggcgcggaattcctcgagctggattttaaagaggaagctggcagcggcgatggctatgccaaagtgatgtcggacgcgttcatcaaagcggaaatggaactctttgccgcccaggcaaaagaggtcgatatcattgtcaccaccgcgcttattccaggcaaaccagcgccgaagctaattacccgtgaaatggttgactccatgaaggcgggcagtgtgattgtcgacctggcagcccaaaacggcggcaactgtgaatacaccgtgccgggtgaaatcttcactacggaaaatggtgtcaaagtgattggttataccgatcttccgggccgtctgccgacgcaatcctcacagctttacggcacaaacctcgttaatctgctgaaactgttgtgcaaagagaaagacggcaatatcactgttgattttgatgatgtggtgattcgcggcgtgaccgtgatccgtgcgggcgaaattacctggccggcaccgccgattcaggtatcagctcagccgcaggcggcacaaaaagcggcaccggaagtgaaaactgaggaaaaatgtacctgctcaccgtggcgtaaatacgcgttgatggcgctggcaatcattctttttggctggatggcaagcgttgcgccgaaagaattccttgggcacttcaccgttttcgcgctggcctgcgttgtcggttattacgtggtgtggaatgtatcgcacgcgctgcatacaccgttgatgtcggtcaccaacgcgatttcagggattattgttgtcggagcactgttgcagattggccagggcggctgggttagcttccttagttttatcgcggtgcttatagccagcattaatattttcggtggcttcaccgtgactcagcgcatgctgaaaatgttccgcaaaaattaaggggtaacatatgtctggaggattagttacagctgcatacattgttgccgcgatcctgtttatcttcagtctggccggtctttcgaaacatgaaacgtctcgccagggtaacaacttcggtatcgccgggatggcgattgcgttaatcgcaaccatttttggaccggatacgggtaatgttggctggatcttgctggcgatggtcattggtggggcaattggtatccgtctggcgaagaaagttgaaatgaccgaaatgccagaactggtggcgatcctgcatagcttcgtgggtctggcggcagtgctggttggctttaacagctatctgcatcatgacgcgggaatggcaccgattctggtcaatattcacctgacggaagtgttcctcggtatcttcatcggggcggtaacgttcacgggttcggtggtggcgttcggcaaactgtgtggcaagatttcgtctaaaccattgatgctgccaaaccgtcacaaaatgaacctggcggctctggtcgtttccttcctgctgctgattgtatttgttcgcacggacagcgtcggcctgcaagtgctggcattgctgataatgaccgcaattgcgctggtattcggctggcatttagtcgcctccatcggtggtgcagatatgccagtggtggtgtcgatgctgaactcgtactccggctgggcggctgcggctgcgggctttatgctcagcaacgacctgctgattgtgaccggtgcgctggtcggttcttcgggggctatcctttcttacattatgtgtaaggcgatgaaccgttcctttatcagcgttattgcgggtggtttcggcaccgacggctcttctactggcgatgatcaggaagtgggtgagcaccgcgaaatcaccgcagaagagacagcggaactgctgaaaaactcccattcagtgatcattactccggggtacggcatggcagtcgcgcaggcgcaatatcctgtcgctgaaattactgagaaattgcgcgctcgtggtattaatgtgcgtttcggtatccacccggtcgcggggcgtttgcctggacatatgaacgtattgctggctgaagcaaaagtaccgtatgacatcgtgctggaaatggacgagatcaatgatgactttgctgataccgataccgtactggtgattggtgctaacgatacggttaacccggcggcgcaggatgatccgaagagtccgattgctggtatgcctgtgctggaagtgtggaaagcgcagaacgtgattgtctttaaacgttcgatgaacactggctatgctggtgtgcaaaacccgctgttcttcaaggaaaacacccacatgctgtttggtgacgccaaagccagcgtggatgcaatcctgaaagctctgtaa |
| *gapN* | atgtttgaaaatatatcatcaaatggagtttataaaaatctatttgatggaaaatgggttgaaagtaagacaaataaaaccatagaaacgcattctccttatgatggaagtttaattggaaaagttcaggccttatcaaaagaggaagttgatgagatttttaaaagttcaagaacagctcagaaaaaatggggtgaaactccaataaatgagcgtgctagaatcatgcgtaaagcagctgatatactagatgataacgcagaatatatagcaaaaattctttcaaatgagatagcaaaagatttaaaatcttctctttcagaagtaaaaagaacagctgattttataagatttacagctaatgaaggtactcatatggaaggagaagctattaactcagataattttcctggttctaaaaaagataaactttctctagttgaaagagttcctttaggaatagttttagctatatctccttttaattatcctgtaaatctttctgggtctaaggttgctccagcacttatagctggaaatagtgttgttttaaaaccttctacaactggtgctataagcgcacttcatcttgcagaaatttttaatgcagctggtcttccagcaggtgttttaaacactgtaacaggaaaagggtctgaaataggcgattatttaattacccatgaagaagtaaactttattaactttacgggaagctctgctgtaggtaagcatatttcaaaaatagctggaatgatacctatggttcttgagcttggtggtaaagatgctgctatagttctcgaagatgccaatcttgaaacaacagctaaaagcatagtatctggagcatatggatactccggccaaaggtgtactgctgtaaaaagagttcttgtaatggataaagtagctgatgaattagttgaacttgttacaaaaaaagttaaagaattaaaggtaggtaatccttttgatgatgttacaataaccccacttatagacaacaaggcagcagattatgttcaaactctcattgacgacgctatcgaaaagggtgcaactcttatcgttggaaataagcgtaaagaaaatttaatgtatcctactttatttgataatgtaactgctgatatgcgtattgcttgggaagaaccatttggaccagttttacctattattcgtgtaaaaagcatggatgaagcaatagaattagcaaatagatctgaatatggtcttcaatctgcagtatttactgaaaatatgcatgatgccttttatattgccaataaattagatgttggaactgttcaagtaaataataagcctgaaagaggcccagatcacttcccattccttggaacaaagtcatcaggtatgggcactcaaggaattcgatacagtatagaggcaatgacaaggcataaatcaatagttttaaacctataa |
| *metK* | atgagtcgccgtctgtttaccagcgaaagtgtgaccgaaggccatccggataaaattgccgatcagattagcgataccattctggatgccctgctgcgcgaagatccgaccagccgtgttgccgtggaaaccctgattaccaccggcctggtgcatgtggccggcgaagttaccaccaaagcatacgctccgattgcccagctggtgcgtgaaaaaattctggaaattggctatgatagtagtaaaaaaggctttgatggtgcaagctgcggcgtgagtgttagtattggtgcccagagcccggatattgcacagggtgttgataccgcatacgaaagtcgtgttgaaggcgatgaagatgaactggatcgccagggcgcaggcgatcagggcctgatgtttggctatgcctgtgatgaaaccccggaactgatgccgctgccgattcatctggcccatcgtctgagccgccgcctgagcgaagtgcgtaaaaatggcaccattccgtatctgcgtccggatggcaaaacccaggtgaccattgaatatgatggtgacaaagcagtgcgtctggataccgttgtggttagcagccagcatgccagtgatattgatctggaaagtctgctggcaccggatattcgtgaatttgttgttgaaccggaactgaaagcactggttgaagatggcattaagctggaaaccgaaggttatcgtctgctggtgaatccgaccggtcgctttgaaattggtggtccgatgggcgatgcaggcctgaccggccgtaaaattattattgatacctatggtggcatgagccgtcatggcggtggcgcctttagcggtaaagatccgagcaaagtggatcgtagtgccgcctatgcaatgcgctgggttgccaaaaatgtggttgcagccggtctggccagtcgctgcgaagtgcaggttgcatacgctattggcaaagcagaaccggttggtctgtttgtggaaacctttggcaccaataccattgataccgataaaattgaacaggccattagcgaagtttttgatctgcgtccggcagcaattattcgcgatctggatctgctgcgtccgatctatagccagaccgcagcatacggtcattttggccgtagtctgccggaattcacttgggaaaaaaccgatcgtgttgatggttgcggtcgcccgccggtttggcgtgctgatctgctgccgctggtgcattaa |

**Table S4** Effect of adding L-Methionine in FA biosynthetic pathway.

p15aT5 refers to JM109(DE3)/p15a-T5-tal-sam5-comt, Control refers to JM109(DE3) /p15a-T5-tal-sam5-comt+pCL1920-T7. The fermentation was carried out in M9Y medium supplemented with 2% (v/v) glycerol and 1 g/L L-Tyrosine, 1g/L L-Methionine at 28°C 250 rpm for 5 days.

| Strains | OD_600_ | FA(mg/L) | CA(mg/L) |
| --- | --- | --- | --- |
| p15aT5 | 8.37±0.24 | 180.50±10.36 | 5.06±0.12 |
| p15aT5/met | 7.88±0.55 | 83.48±7.04 | 3.78±0.05 |
| Control | 6.60±0.32 | 178.27±1.79 | 15.39±0.6 |
| Control/met | 7.16±0.32 | 116.46±7.51 | 3.84±0.02 |


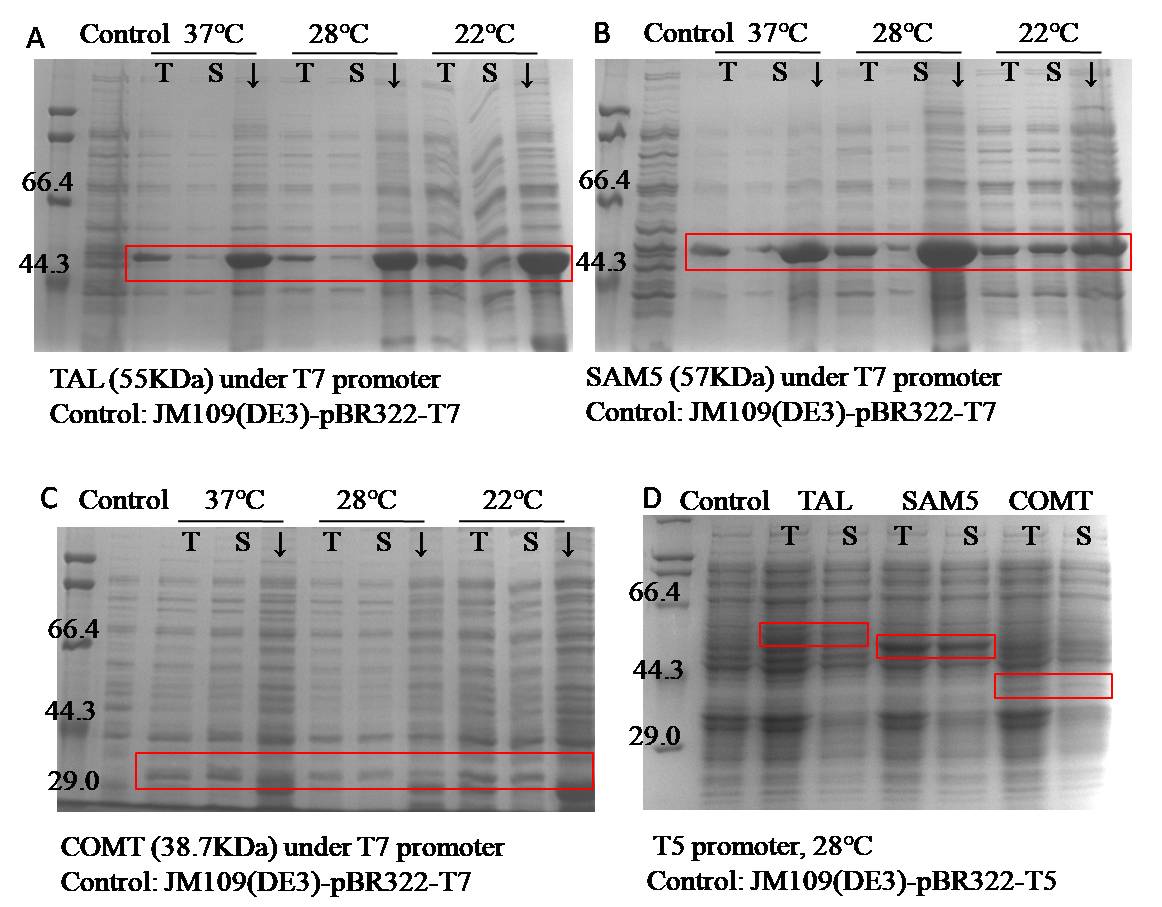


**Fig. S1** Sodium dodecyl sulfate polyacrylamide gel electrophoresis results of TAL, SAM5 and COMT expression

(A-C) SDS-PAGE of TAL, SAM5 and COMT protein expression in *E.coi* JM109(DE3) under T7 promoter control at 37 °C, 28 °C and 22 °C. Protein expression was induced after 37 °C, 250 rpm cultivation in LB medium for 2 h, by adding 0.1 mM IPTG, for 3, 5 and 12 hours respectively. Cells were collected and prepared samples of total protein (T), soluble protein (S), and protein in sediment (↓).

(D) SDS-PAGE of TAL, SAM5 and COMT protein expression in *E.coi* JM109(DE3) under T5 promoter control at 28 °C. Protein expression was induced after 37 °C, 250 rpm cultivation in LB medium for 2 h, by adding 0.1 mM IPTG, for 5 hours. Cells were collected and prepared samples of total protein (T) and soluble protein (S).

**References**

J. Overhage, A. Steinbuchel, H. Priefert (2003) Highly Efficient Biotransformation of Eugenol to Ferulic Acid and Further Conversion to Vanillin in Recombinant Strains of *Escherichia coli*. Applied and Environmental Microbiology 69(11): 6569-6576.

O. Choi, C.-Z. Wu, S.Y. Kang, J.S. Ahn, T.-B. Uhm, Y.-S. Hong (2011) Biosynthesis of plant-specific phenylpropanoids by construction of an artificial biosynthetic pathway in *Escherichia coli*. Journal of Industrial Microbiology & Biotechnology 38(10): 1657-1665.

S.-Y. Kang, O. Choi, J.K. Lee, B.Y. Hwang, T.-B. Uhm, Y.-S. Hong (2012) Artificial biosynthesis of phenylpropanoic acids in a tyrosine overproducing *Escherichia coli* strain. Microbial Cell Factories 11(1): 153.

J. Ni, F. Tao, H. Du, P. Xu (2015) Mimicking a natural pathway for de novo biosynthesis: natural vanillin production from accessible carbon sources. Sci Rep 5: 13670.

S. Wang, S. Zhang, A. Xiao, M. Rasmussen, C. Skidmore, J. Zhan (2015) Metabolic engineering of *Escherichia coli* for the biosynthesis of various phenylpropanoid derivatives. Metab Eng 29: 153-159.

J.L. Rodrigues, D. Gomes, L.R. Rodrigues (2020) A Combinatorial Approach to Optimize the Production of Curcuminoids From Tyrosine in *Escherichia coli*. Front Bioeng Biotechnol 8: 59.
